# Our Editorial Space

**Published:** 1873-08

**Authors:** 


### PART III.

### EDITORIAL AND MISCELLANEOUS.

OUR THANKS are due those of our subscribers who have so promptly responded to our demands upon them in paying their subscriptions. We know those who have not done so have satisfactory reasons for the delay, and it will only be necessary on our part to remind them of *our* wants to have them come promptly forward. So many medical journals in the South have failed, that it is impossible to have one published without some one becoming responsible to the publisher, or indorsing for each of the subscribers. This we have done, as we have no fear—nor would we have any trouble, if the matter could be stated as it is to each of them. They can, therefore, appreciate our relations to them and the publisher. The latter demands so much each month for publication. Our friends, when aware of this, and who still owe us, will see their duty in its true light, and, we doubt not, cheerfully and promptly forward the small amounts due for subscription. To save expense, we will not in future send bills, but will place a red cross as a reminder on the journal of those who have forgotten to remit the subscription price. If we should fail to credit them in the following issue of the journal, they will please inform us.

OUR EDITORIAL SPACE, as will be seen, is filled almost exclusively by correspondence. It will be remembered that we have, time and again, solicited our friends to thus use our columns for the general weal of the profession. We are glad to have them do so. The RECORD is the journal of the profession, and is open to all honorable gentlemen who may desire to discuss subjects for the general welfare. We, therefore, again invite our friends to send us short paragraphs for the special object of doing good and advancing the interests of a common cause. Let the profession be degraded, and all are degraded with it. If the profession, by union—which is strength—is elevated, then each individual is elevated, and his best interests secured and protected.

